# Selenium-induced metabolic reprogramming in soybean activates nucleotide transport pathways and suppresses primary carbon metabolism

**DOI:** 10.1038/s41598-026-57640-w

**Published:** 2026-06-16

**Authors:** Dezhi Han, Wei Li, Shuang Zhang, Honglei Ren, Qi Zhang, Jun Jin, Zhaoji Jiang, Hua Qian, Jidao Du

**Affiliations:** 1 College of Agriculture, Heilongjiang Bayi Agricultural University, Daqing, 163000 China; 2 Heilongjiang Academy of Agricultural Sciences, Harbin, 150086 China; 3 Heilongjiang University of Science and Technology, Harbin, 150020 China

**Keywords:** Soybean, Selenium biofortification, Nucleotide transmembrane transport, ABC transporters, Transcriptome, GSVA, Antioxidant enzymes, Metabolic reallocation, Biochemistry, Biotechnology, Molecular biology, Physiology, Plant sciences

## Abstract

Selenium biofortification in soybean is one approach to addressing widespread selenium deficiency in human populations, yet the temporal dynamics of gene expression and antioxidant responses following selenium enrichment remain poorly characterized. Here, we examined the physiological and transcriptional responses of the HK88 soybean variety to selenium-enriched nutrient solution treatment, with seedlings sampled at 1, 24, and 48 h post-treatment. Total tissue selenium rose from 0.01 mg/kg at 1 h to 0.33 mg/kg at 48 h, with parallel increases in both inorganic and organic fractions, consistent with active biotransformation, though this interpretation remains to be confirmed experimentally. Antioxidant responses followed a distinct temporal pattern: superoxide dismutase (SOD) and peroxidase (POD) activities were initially lower in treated plants at 1 h relative to controls but were elevated at 24 and 48 h, while catalase (CAT) activity remained comparatively low across all time points. Malondialdehyde (MDA) levels were lower in selenium-treated plants at 1 h, suggesting early membrane stabilization, though this difference was no longer apparent by 48 h. RNA sequencing of 18 libraries identified 6,793 differentially expressed genes (DEGs) at 1 h, peaking at 13,196 (approximately 18% of the 72,513 annotated genes) at 24 h, then declining to 8,996 at 48 h. A Venn diagram analysis identified 565 DEGs shared across all three time points, comprising 196 consistently up-regulated and 369 consistently down-regulated genes. Up-regulated genes were enriched for nucleotide transmembrane transport functions, including ATP, ADP, and purine transport, with associated ABC transporter activity. Down-regulated genes were predominantly associated with primary carbon metabolism, including monosaccharide biosynthesis, gluconeogenesis, and the Calvin cycle. Gene Set Variation Analysis (GSVA) indicated positive enrichment scores for nucleotide transport pathways at 24 and 48 h in treated plants, contrasting with negative scores in controls. Mantel tests revealed significant associations between gene set activity profiles and measured physiological traits, particularly for gene sets related to molecular function and selenium accumulation. These findings suggest that selenium biofortification in HK88 is associated with a coordinated metabolic shift, in which primary carbon fixation is reduced while nucleotide transport capacity is enhanced, supporting antioxidant defense during selenium assimilation.

**Supplementary Information:**

The online version contains supplementary material available at https://doi.org/10.1038/s41598-026-57640-w.

## Introduction

Selenium (Se) is a trace element required by humans and animals, where it is incorporated into selenoproteins that participate in redox homeostasis, thyroid hormone metabolism, and immune function^1,2^. The Chinese Nutrition Society recommends a minimum daily intake of 50 μg, while the International Society for Selenium Research suggests 60 μg^3^. Evidence also indicates that intakes of approximately 200 μg per day may be associated with reduced disease risk^4^, though intakes exceeding 400 μg daily carry potential health risks^5^. Approximately one billion people worldwide experience some degree of selenium deficiency^6^, which has been associated with conditions including Keshan disease, myocardial degeneration, hepatitis, cataracts, and multiple sclerosis^7^. Plants constitute the primary dietary source of selenium and can convert inorganic environmental selenium into organic forms, facilitating its entry into the food chain^8^. Dietary selenium intake therefore depends substantially on the selenium content of crop plants, which in turn reflects the bioavailability of selenium in agricultural soils^9^. Agricultural biofortification, the deliberate enhancement of selenium content in edible plant tissues, has been proposed as a practical strategy for addressing selenium insufficiency at the population level^10^.

Soybean (*Glycine max* L. Merr.) is an economically important legume crop, contributing high-quality protein and oil to human diets and animal feed worldwide^11,12^. Its capacity to accumulate selenium makes it a candidate for selenium biofortification programs^13,14^. Exogenous selenium supply, whether through soil amendment, foliar application, or nutrient solution treatment, increases selenium concentrations in soybean tissues, with a substantial proportion converted from inorganic selenate or selenite into organic forms, principally selenomethionine^15^. Organic selenium forms are generally more efficiently absorbed and utilized by humans than inorganic selenium, which supports the agronomic and nutritional rationale for selenium-enriched soybean^14^.

Beyond its nutritional role, selenium may also confer direct physiological benefits to plants, particularly under stress conditions. At low to moderate concentrations, selenium has been reported to stimulate growth, enhance antioxidant enzyme activities, and reduce the accumulation of reactive oxygen species (ROS)^16,17^. The antioxidant enzyme network comprising SOD, POD, and CAT constitutes a primary enzymatic defense against oxidative damage, and induction of this network by selenium has been documented across several crop species^18,19^. Selenium supplementation has also been associated with reduced malondialdehyde (MDA) accumulation, a marker of lipid peroxidation, and with stabilization of membrane integrity under stress^17^. However, the temporal dynamics of these antioxidant responses and the underlying molecular mechanisms remain incompletely characterized in soybean.

Transcriptomic approaches have provided insights into the molecular basis of selenium responses in crops. RNA-seq studies in wheat (*Triticum aestivum*) and tea (*Camellia sinensis*) have identified thousands of selenium-responsive DEGs spanning metabolic, transport, and stress-response pathways^20,21^. In soybean, ABC transporters have been implicated in selenium uptake and compartmentalization, and transcription factor networks appear to contribute to the coordination of selenium metabolism with broader stress-adaptation programs^22,23^. Nevertheless, a time-resolved, integrative analysis linking gene expression dynamics to quantitative physiological measurements at multiple time points has not been established for soybean under selenium enrichment conditions.

The HK88 soybean variety, developed and characterized by our group for adaptation to diverse environments in northern China, was selected for this study based on preliminary observations of relatively high selenium retention under supplementation conditions. Whether HK88 constitutes a formally validated selenium-accumulating variety relative to other cultivars remains to be established in dedicated comparative studies; the present work aims to characterize its physiological and transcriptional responses to selenium enrichment rather than to make comparative claims. Seedlings were exposed to a selenium-enriched nutrient solution and sampled at 1, 24, and 48 h. We measured total, inorganic, and organic selenium fractions, assessed antioxidant enzyme activities (SOD, POD, CAT), and quantified MDA content. RNA-seq profiling was conducted on 18 samples, with three biological replicates per group at each time point. Integrating differential expression analysis, GO and KEGG enrichment, GSVA, and Mantel correlation tests, we constructed a framework linking transcriptional changes to selenium accumulation and antioxidant physiology in HK88.

## Materials and methods

### Plant material and selenium treatment

HK88 soybean seedlings (obtained from the Heilongjiang Bayi Agricultural University seed collection, Daqing, China) were grown under controlled conditions in a growth chamber (25°C day/20°C night; 16-h photoperiod; 60–70% relative humidity) in sterilized vermiculite:perlite (3:1, v/v). Seedlings at the V2 growth stage were treated with either a selenium-enriched nutrient solution (Na2SeO4 in Hoagland solution at 20 µM; treatment group, P) or standard Hoagland solution alone (control group, Q). Tissue samples were harvested at 1, 24, and 48 h post-treatment, designated P1/Q1, P4/Q4, and P5/Q5, respectively. Fresh tissue samples (0.1 g) were immediately ground into a fine powder for downstream assays.

### Determination of selenium fractions

Total, inorganic, and organic selenium concentrations were measured by hydride-generation atomic fluorescence spectrometry following established protocols. Tissue samples were acid-digested, and selenium fractions were assessed spectrophotometrically. Total selenium was quantified after complete reduction of all selenium forms to Se(IV), inorganic selenium was measured directly, and organic selenium was calculated by subtraction. Three biological replicates were analyzed per treatment and time point.

### Antioxidant enzyme activity and lipid peroxidation assays

Frozen tissue samples were homogenized in ice-cold phosphate buffer (pH 7.8) containing EDTA and polyvinylpolypyrrolidone, and supernatants were obtained by centrifugation at 12,000 × g for 20 min at 4°C. SOD activity (EC 1.15.1.1) was assessed by measuring inhibition of nitro blue tetrazolium photoreduction at 560 nm. POD activity (EC 1.11.1.7) was determined spectrophotometrically at 470 nm by monitoring guaiacol oxidation in the presence of H_2_O_2_. CAT activity (EC 1.11.1.6) was measured by the decrease in absorbance at 240 nm during H2O2 decomposition. MDA concentrations were quantified using the thiobarbituric acid reactive substances (TBARS) assay, with absorbance readings at 532 nm and 600 nm to correct for non-specific turbidity. All values were expressed per gram of fresh tissue.

### RNA extraction and quality assessment

Total RNA was extracted from all 18 tissue samples using TRIzol reagent (Invitrogen, CA, USA) according to the manufacturer’s instructions. RNA integrity and quantity were assessed by 1.2% agarose gel electrophoresis and an Agilent 2100 Bioanalyzer (Agilent Technologies, Santa Clara, CA, USA). Only samples with an RNA Integrity Number (RIN) above 7.0 were used for library preparation.

### cDNA library construction and RNA sequencing

Strand-specific cDNA libraries were prepared using the NEBNext Ultra™ RNA Library Prep Kit for Illumina (NEB, Beijing, China). Briefly, mRNA was isolated from total RNA using oligo(dT) magnetic beads, fragmented, and reverse transcribed. The resulting double-stranded cDNA was end-repaired, A-tailed, ligated to indexed adapters, size-selected, and PCR-amplified. Library quality was verified using a Bioanalyzer, and libraries were pooled at equimolar concentrations. Sequencing was performed on an Illumina NovaSeq 6000 platform (Illumina Inc., California, USA) using 150-bp paired-end reads. Raw sequencing data have been deposited in the NCBI Sequence Read Archive under BioProject accession number PRJNA1427924.

### Read processing and genome alignment

Sequencing reads were processed to remove adapter sequences, low-quality bases, and contaminants using standard quality-control parameters. Cleaned reads were aligned to the Glycine max reference genome (GCF_004193775.1, Wm82.a4.v1; https://www.ncbi.nlm.nih.gov/datasets/genome/GCF_004193775.1) using STAR (v2.7.10a) with default settings. Alignment quality was evaluated using mapping rate, unique mapping rate, and multi-mapping rate. Only uniquely aligned reads were retained for downstream expression quantification.

### Gene expression quantification

Gene-level expression was quantified using RNA-Seq by Expectation–Maximization (RSEM v1.3.2; http://deweylab.biostat.wisc.edu/rsem/). Expression levels were normalized as transcripts per kilobase of exon model per million mapped reads (TPM) to account for differences in gene length and sequencing depth.

### Differential expression analysis

Differential gene expression between treatment and control groups at each time point (P1 vs. Q1, P4 vs. Q4, P5 vs. Q5) was assessed using the edgeR package in R. Raw count data were normalized using the trimmed mean of M-values (TMM) method. Differential expression was tested using quasi-likelihood F-tests, and p-values were adjusted for multiple testing using the false discovery rate (FDR) method. Genes with FDR < 0.05 and a nominal *p*-value < 0.05 were classified as differentially expressed genes (DEGs). To identify hub genes with large expression changes, a more stringent threshold of |log₂ fold-change|> 12 and FDR < 0.01 was applied.

### Functional enrichment analysis

Gene Ontology (GO; http://geneontology.org/) and Kyoto Encyclopedia of Genes and Genomes (KEGG; https://www.genome.jp/kegg/) pathway enrichment analyses were conducted on DEG sets using the clusterProfiler package (v4.8.2) in R, with Benjamini-Hochberg (BH) correction applied for multiple comparisons. KEGG pathway map (map04010) reproduced with permission from Kanehisa Laboratories (Ref. 260,496; CC BY 4.0); source acknowledged per Kanehisa and Goto^24–26^. Terms with an adjusted p-value below 0.05 were considered significantly enriched; a stricter threshold of *p*.adjust < 0.01 was used for core analyses. Fold enrichment was calculated as GeneRatio/BgRatio, where GeneRatio is the proportion of DEGs annotated to a given term, and BgRatio is the proportion of all annotated genes assigned to that term. Transcription factor enrichment analysis was performed using STAMP software with FDR correction.

### Gene set variation analysis

Gene Set Variation Analysis (GSVA) was applied to evaluate pathway-level activity across samples. GSVA is a non-parametric, unsupervised method that converts a gene-by-sample expression matrix into a pathway-by-sample enrichment score matrix. Gene sets were derived from significantly enriched GO and KEGG terms identified in the DEG analysis. Enrichment scores, which range from negative (pathway suppression) to positive (pathway activation), were used to assess functional differences between treatment and control groups at each time point, and group differences in GSVA scores were tested for statistical significance.

### Correlation analysis

Associations between gene set activity profiles (derived from GSVA) and physiological variables (SOD, POD, CAT, MDA, total Se, inorganic Se, organic Se) were assessed using partial Mantel tests and Pearson correlation. Mantel tests evaluated correlations between multivariate distance matrices representing gene set categories (Biological Process, Molecular Function, Cellular Component, and KEGG) and the physiological trait matrix, with significance assessed by permutation testing. Pearson correlation coefficients were calculated between individual GSVA scores for each enriched term and each physiological variable across all samples, with p-values adjusted using the Benjamini–Hochberg procedure.

### Statistical analysis and data visualization

For physiological measurements, statistical comparisons between treatment and control groups at each time point were performed using two-tailed t-tests. Data are presented as means ± standard error of three biological replicates. All transcriptomic analyses, statistical testing, and data visualization were performed in R (version 4.2.1 or higher) using packages including ggplot2, ComplexHeatmap, VennDiagram, and custom scripts.

## Results

### Selenium accumulation in HK88 soybean increases progressively with treatment duration

**Fig. 1:**
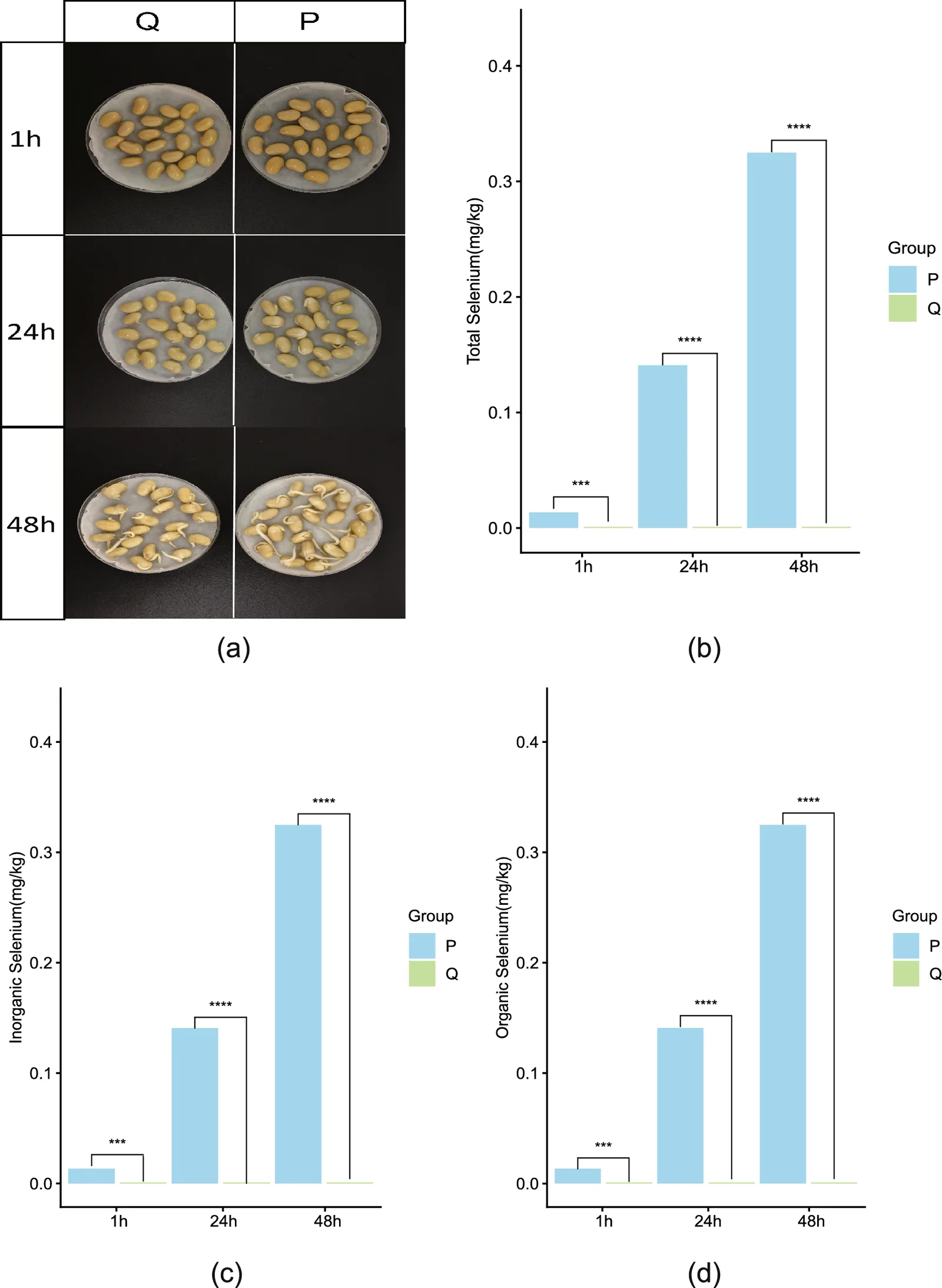
Selenium accumulation in HK88 soybean seedlings. (**a**) Experimental design. Treatment and control groups are labeled “P” and “Q”, respectively. Concentrations of total (**b**), inorganic (**c**), and organic (**d**) selenium were determined at 1 h (P1/Q1), 24 h (P4/Q4), and 48 h (P5/Q5) post-treatment.

To characterize the dynamics of selenium uptake in HK88, seedlings were treated with a selenium-enriched nutrient solution (group P) or standard Hoagland solution (control group Q) and sampled at 1, 24, and 48 h post-treatment (P1/Q1, P4/Q4, and P5/Q5, respectively; Fig. 1a). For transcriptomic profiling, 18 RNA-seq libraries were constructed, yielding 40.3–49.9 million 150-bp paired-end raw reads per library (Table S1). After quality filtering, high-quality base proportions (Q20: 99.31–99.65%; Q30: 97.25–98.61%) and GC content (43.43–45.06%) were consistent across all samples (Table S1). Alignment to the Glycine max reference genome (GCF_004193775.1) achieved an average unique mapping rate of 94.72% (range: 90.90–97.23%) with a mismatch rate of ≤ 0.24% per base across all 18 libraries (Table S2). A total of 72,513 genes were identified in the reference genome.

Total selenium content in P-group plants increased significantly over 48 h, rising from 0.01 mg/kg at 1 h to 0.33 mg/kg at 48 h, while control plants maintained near-baseline levels throughout (Fig. 1b). This accumulation was observed in both inorganic and organic fractions, each reaching approximately 0.32–0.33 mg/kg by 48 h in treated seedlings (Fig. 1c,d). The parallel increase in both fractions is consistent with active biotransformation during the treatment period, though this interpretation cannot be confirmed from accumulation data alone. Organic selenium in particular rose from 0.02 mg/kg at 1 h to 0.33 mg/kg at 48 h, consistent with metabolic conversion of inorganic selenate or selenite into organic selenoamino acids. These accumulation patterns suggest that HK88 may serve as a suitable experimental model for investigating the molecular mechanisms of selenium biofortification, though comparative studies with other varieties would be needed to confirm its status as a high-accumulating genotype.

### Selenium treatment induces a temporally ordered antioxidant response

To characterize physiological responses to selenium enrichment, we measured the activities of SOD, POD, and CAT, and quantified MDA as an indicator of lipid peroxidation. The observed changes were stage-dependent and did not follow a straightforward pattern of uniform stress-triggered enzyme induction.

**Fig. 2:**
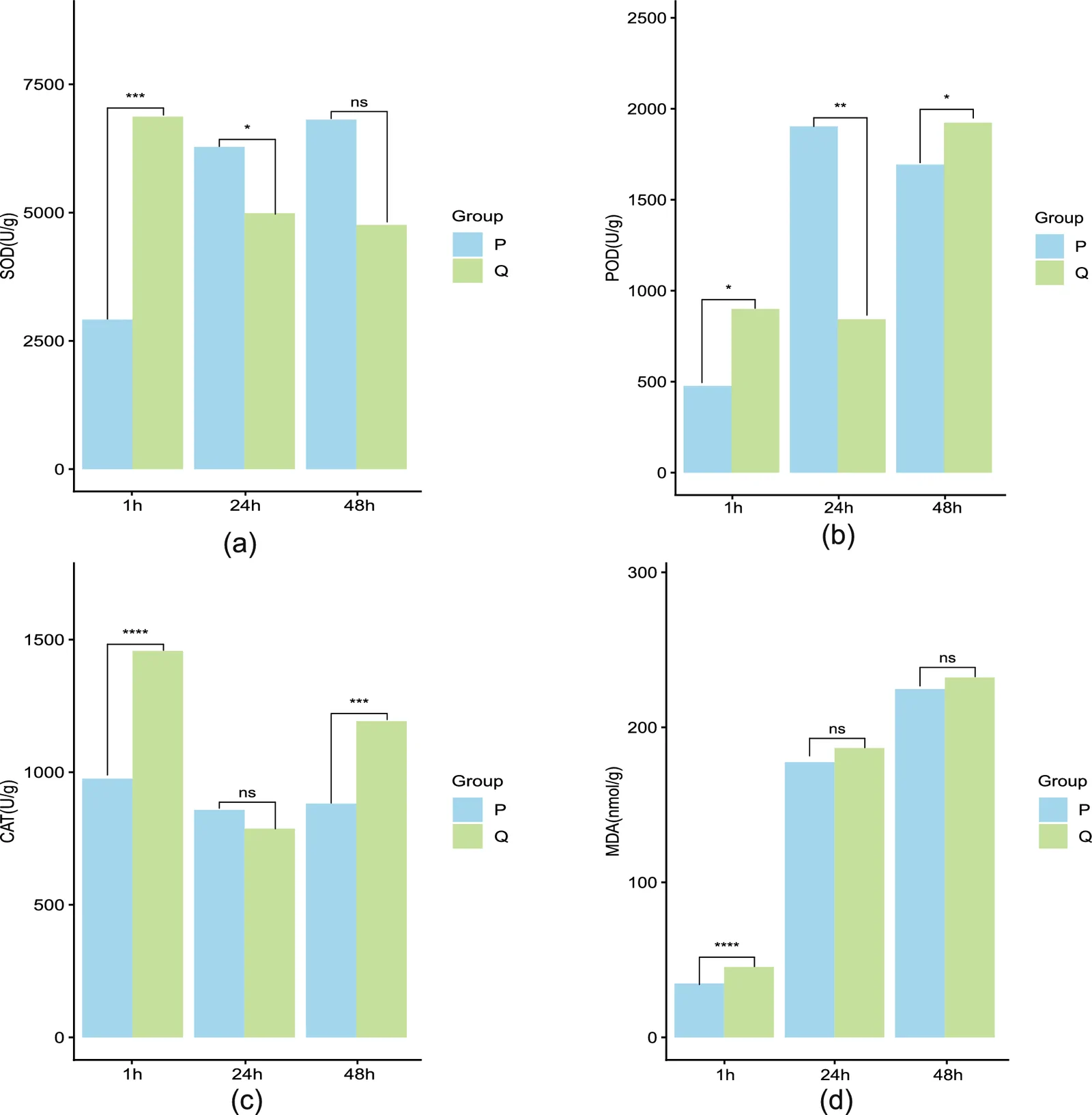
Dynamic changes in physiological indices in HK88 soybean. Measurements were made at 1 h (P1/Q1), 24 h (P4/Q4), and 48 h (P5/Q5) post-treatment. (**a**) SOD activity. (**b**) POD activity. (**c**) CAT activity. (**d**) MDA concentration. Asterisks indicate statistically significant differences between treatment and control (t-test).

SOD activity was lower in selenium-treated seedlings at 1 h (approximately 2,800 vs. 7,000 U/g in controls), but this relationship reversed by 24 h, when plants treated showed higher activity (approximately 6,500 U/g) than controls (approximately 5,000 U/g). At 48 h, treated plants maintained elevated SOD activity (approximately 7,000 U/g) relative to controls (approximately 4,800 U/g), though the difference was reduced (Fig. 2a). This biphasic pattern may reflect an initial phase of metabolic adjustment during early selenium assimilation, followed by enhanced superoxide-scavenging capacity as selenium metabolism is established.

POD activity followed a comparable trajectory. At 1 h, control plants showed approximately double the POD activity of selenium-treated seedlings (900 vs. 450 U/g), while by 24 h this relationship had reversed, with treated plants reaching approximately 1,900 U/g compared to 850 U/g in controls (Fig. 2b). This represents a substantial induction of peroxidase activity in treated plants over the 1–24-h interval. By 48 h, POD activity in treated plants had declined modestly to approximately 1,650 U/g, while controls rose to approximately 1,900 U/g, suggesting convergence toward similar activity levels as selenium assimilation progressed.

CAT activity was consistently lower in selenium-treated plants across all time points. Controls showed higher CAT activity at 1 h (approximately 1,450 vs. 1,000 U/g), with values narrowing at 24 h (approximately 870 vs. 760 U/g) and diverging again at 48 h (approximately 1,200 vs. 870 U/g; Fig. 2c). The sustained difference in CAT response relative to SOD and POD suggests that selenium enrichment may differentially regulate peroxisomal and cytosolic antioxidant pathways. Since CAT primarily scavenges H2O2 in peroxisomes during photorespiration, while POD acts in the cytosol and cell wall, the preferential induction of POD may reflect the subcellular localization of selenium-induced ROS during uptake and assimilation.

MDA levels in selenium-treated seedlings were lower than in controls at 1 h (approximately 35 vs. 50 nmol/g), suggesting early membrane stabilization despite the observed initial suppression of SOD and POD activities (Fig. 2d). This pattern is consistent with the possibility that selenium may stabilize membranes through mechanisms that are at least partly independent of enzymatic ROS scavenging, though this interpretation requires further experimental support. By 24 h, MDA had increased to similar levels in both groups (approximately 180–190 nmol/g), a pattern that persisted at 48 h (approximately 225–230 nmol/g). The convergence of MDA values between groups, despite maintained differences in enzyme activity, may indicate the establishment of a redox state in which both groups manage oxidative load through distinct mechanisms.

Collectively, these data suggest a staged antioxidant response in which early membrane stabilization precedes the enzyme-level induction of SOD and POD, while CAT remains comparatively suppressed throughout. The delay between selenium exposure and enzyme activity increases is consistent with transcriptional regulation.

### Transcriptomic profiling reveals large-scale, time-dependent differential gene expression

**Fig. 3:**
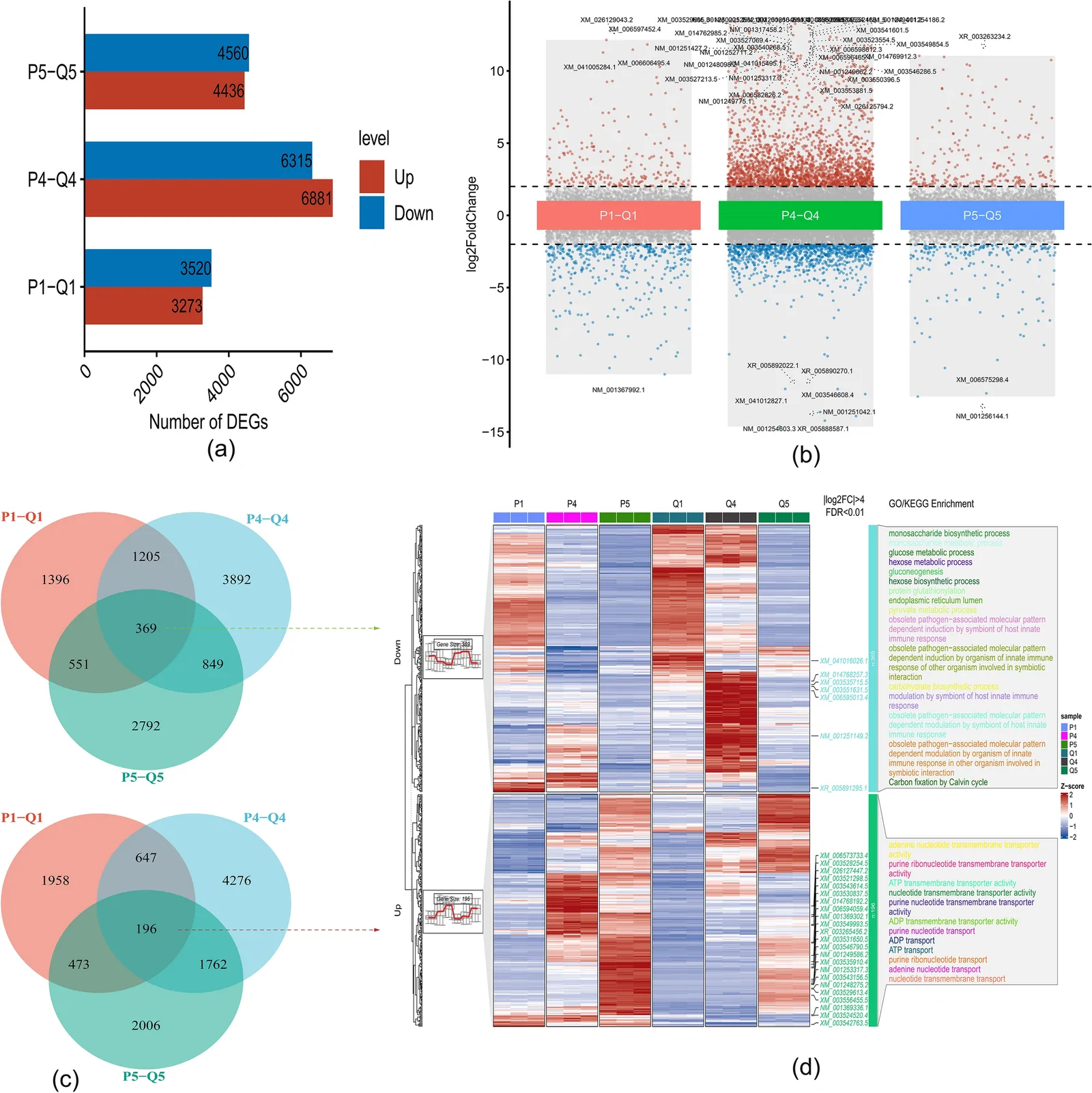
Temporal analysis of differentially expressed genes following selenium-enriched nutrient solution treatment. (**a**) DEG counts between groups P and Q at three time points. (**b**) Volcano plots showing up- (red) and down-regulated (green) genes (|log2FC|> 1, FDR < 0.05); 34 hub genes with |log2FC|> 12 and FDR < 0.01 are shown at the plot extremes. (**c**) Venn diagrams of overlapping DEGs among the three comparison groups; 196 DEGs were up-regulated and 369 down-regulated in P relative to Q across all time points. (**d**) Heatmap of the 565 common DEGs, showing distinct clustering of treatment and control samples.

RNA-seq analysis identified 6,793 DEGs at 1 h (3,273 up-regulated, 3,520 down-regulated), rising to 13,196 at 24 h (6,881 up-regulated, 6,315 down-regulated), and declining to 8,996 at 48 h (4,436 up-regulated, 4,560 down-regulated; Fig. 3a). The peak at 24 h, encompassing approximately 18% of the 72,513 annotated reference genes, indicates that the most extensive transcriptional response to selenium enrichment occurs at this intermediate time point rather than immediately following treatment or at 48 h.

Volcano plots of the three pairwise comparisons showed the distribution of fold-change magnitudes and significance across the transcriptome (Fig. 3b). Most DEGs showed modest changes (|log2FC|< 2), but a subset displayed large expression shifts. Applying a threshold of |log2FC|> 12 and FDR < 0.01 identified hub genes with near-binary expression changes: 2 at 1 h (including XM_026129043.2 at log2FC = 12.145), 26 at 24 h (including NM_001362622.1 at log2FC = 14.339 and XM_014774790.3 at − 14.351), and 6 at 48 h (including NM_001348940.1 at -15.639). The concentration of 26 of the 34 hub genes at 24 h is consistent with this time point representing the peak of transcriptional perturbation.

Venn diagram analysis identified genes with consistent regulation across all three time points (Fig. 3c). Among down-regulated genes, 369 were suppressed at 1, 24, and 48 h, while pairwise overlaps were larger: 1,205 genes at 1 h ∩ 24 h, 849 at 24 h ∩ 48 h, and 551 at 1 h ∩ 48 h. Time-specific down-regulation was also evident (1,396 genes at 1 h; 3,892 at 24 h; 2,792 at 48 h), indicating that selenium-induced suppression involves both a stable core response and stage-specific changes. Up-regulated genes showed comparable patterns: 196 DEGs were consistently induced across all time points, with pairwise overlaps of 647 (1 h ∩ 24 h), 1,762 (24 h ∩ 48 h), and 473 (1 h ∩ 48 h), plus stage-specific induction of 1,958 (1 h), 4,276 (24 h), and 2,006 (48 h) genes. The 565 common DEGs (369 down-regulated, 196 up-regulated) represent a stable transcriptional signature of selenium enrichment in HK88, with expression shifts that are maintained across the 48-h observation period. Hierarchical clustering of these genes across 18 samples separated treatment from control groups, with P-group samples (P1, P4, P5) forming a distinct cluster from Q-group samples (Q1, Q4, Q5; Fig. 3d). The consistency of expression patterns across biological replicates at all three time points supports the reproducibility of these responses.

Functional enrichment of the 565 common DEGs revealed a clear distinction between up- and down-regulated gene sets. Down-regulated genes were associated with carbohydrate metabolism and carbon fixation, including GO terms related to monosaccharide biosynthesis, gluconeogenesis, and carbohydrate biosynthesis, as well as the KEGG pathway for carbon fixation via the Calvin cycle. Up-regulated genes were predominantly associated with nucleotide transmembrane transport, including adenine, purine, ATP, ADP, and purine ribonucleotide transport.

These findings suggest that selenium enrichment in HK88 may involve a reallocation of metabolic resources, with a reduction in transcriptional support for photosynthetic carbon fixation alongside increased capacity for nucleotide transport. The latter is broadly consistent with the involvement of ABC transporters, which are known to facilitate transmembrane transport of nucleotides and metabolites and have been implicated in selenium handling in plants. This apparent metabolic shift was persistent across the 1-to-48-h observation window, suggesting it reflects a stable aspect of the cellular response to selenium rather than a transient adjustment.

### Functional enrichment identifies transmembrane transport and primary carbon metabolism as core-regulated pathways

**Fig. 4:**
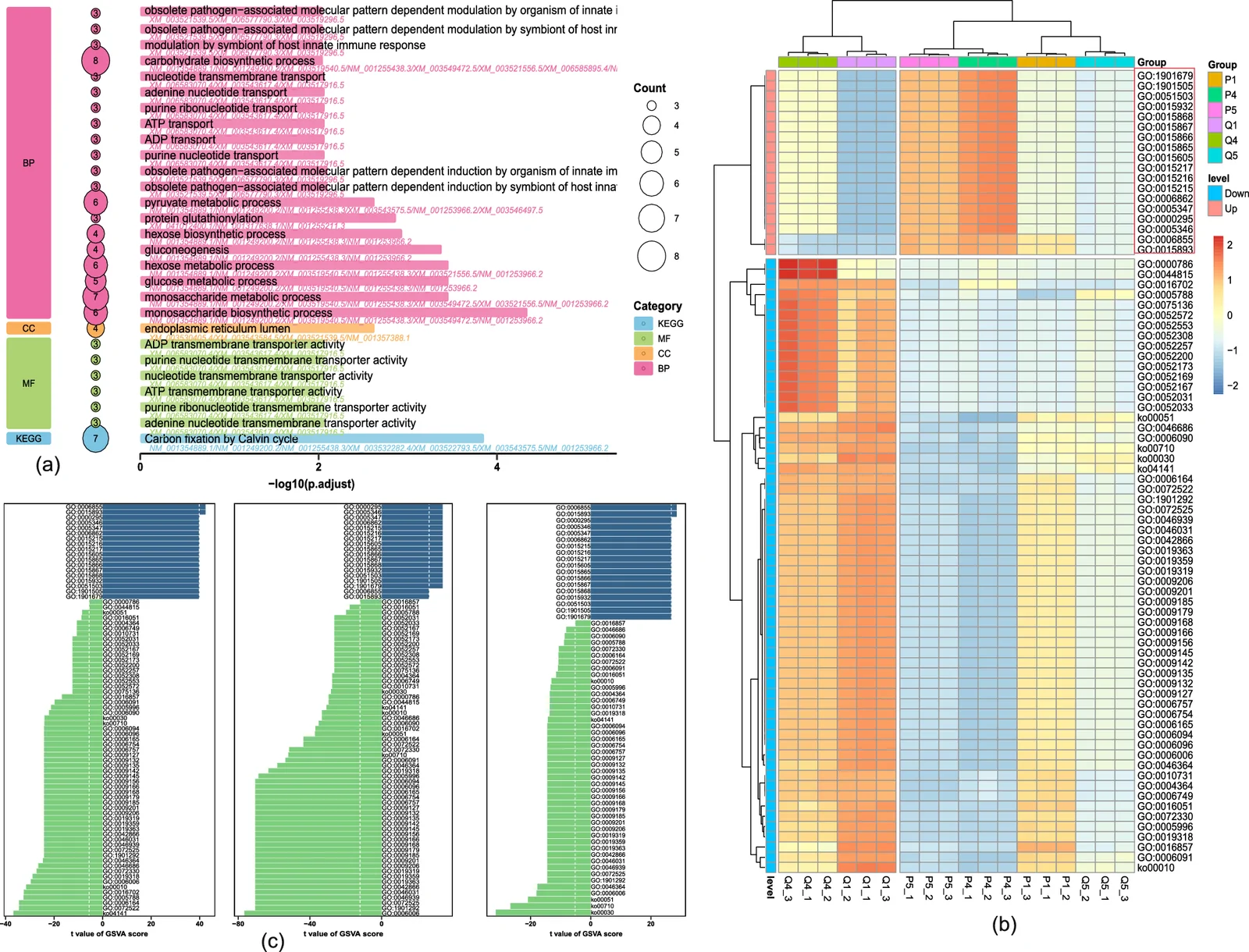
Functional Enrichment of the 565 Common DEGs. (**a**) GO and KEGG enrichment results. Terms are colored by category: Biological Process (pink), Molecular Function (light green), Cellular Component (orange), and KEGG (blue). Bubble size indicates gene count. KEGG pathway map (map04010) reproduced with permission from Kanehisa Laboratories (Ref. 260,496; CC BY 4.0); source acknowledged per Kanehisa and Goto. (**b**) GSVA enrichment scores for 78 enriched terms across samples. Positive scores indicate pathway activation; negative scores indicate suppression. (**c**) Differential GSVA scores between treatment and control at each time point; terms significant at *p* < 0.01 are marked in blue and green.

To identify pathways potentially associated with the response to selenium enrichment, GO and KEGG enrichment analyses were performed on the 565 common DEGs. A total of 73 GO terms and 5 KEGG pathways were significantly enriched (*p*.adjust < 0.05), and 28 terms (27 GO, 1 KEGG) met the stricter threshold of *p*.adjust < 0.01 (Fig. 4a). Among Biological Process terms, 20 were significantly enriched, including those associated with protein glutathionylation (GO:0,010,731), monosaccharide biosynthesis (GO:0,046,364), gluconeogenesis (GO:0,006,094), ATP transport (GO:0,015,867), ADP transport (GO:0,015,866), purine nucleotide transport (GO:0,015,865), nucleotide transmembrane transport (GO:1,901,679), and pyruvate metabolic process (GO:0,006,090), among others. One Cellular Component term was significantly enriched: endoplasmic reticulum lumen (GO:0,005,788). Six Molecular Function terms related to transmembrane transporter activity were enriched, covering adenine nucleotide (GO:0,000,295), purine ribonucleotide (GO:0,005,346), ATP (GO:0,005,347), nucleotide (GO:0,015,215), purine nucleotide (GO:0,015,216), and ADP (GO:0,015,217) transmembrane transporter activity. The single enriched KEGG pathway was carbon fixation by the Calvin cycle (ko00710).

GSVA was then applied to examine the activity of 78 significant pathways derived from the 565 common DEGs across three time points. Of these, 18 GO terms showed positive enrichment scores predominantly in P4 and P5 samples (Fig. 4b). All 18 terms showed statistically significant differences between treatment and control groups (t-values > 10, *p* < 0.01), consistent with the activation of these pathways following selenium-enriched nutrient solution treatment (Fig. 4c). The enriched terms were concentrated in nucleotide and purine transmembrane transport functions, which are commonly associated with ABC transporter activity. These findings suggest that this protein superfamily may participate in the metabolic response to selenium enrichment in HK88, though direct experimental evidence would be required to confirm this hypothesis.

### Activated nucleotide transmembrane transport gene sets correlate positively with selenium accumulation and antioxidant enzyme activities

**Fig. 5:**
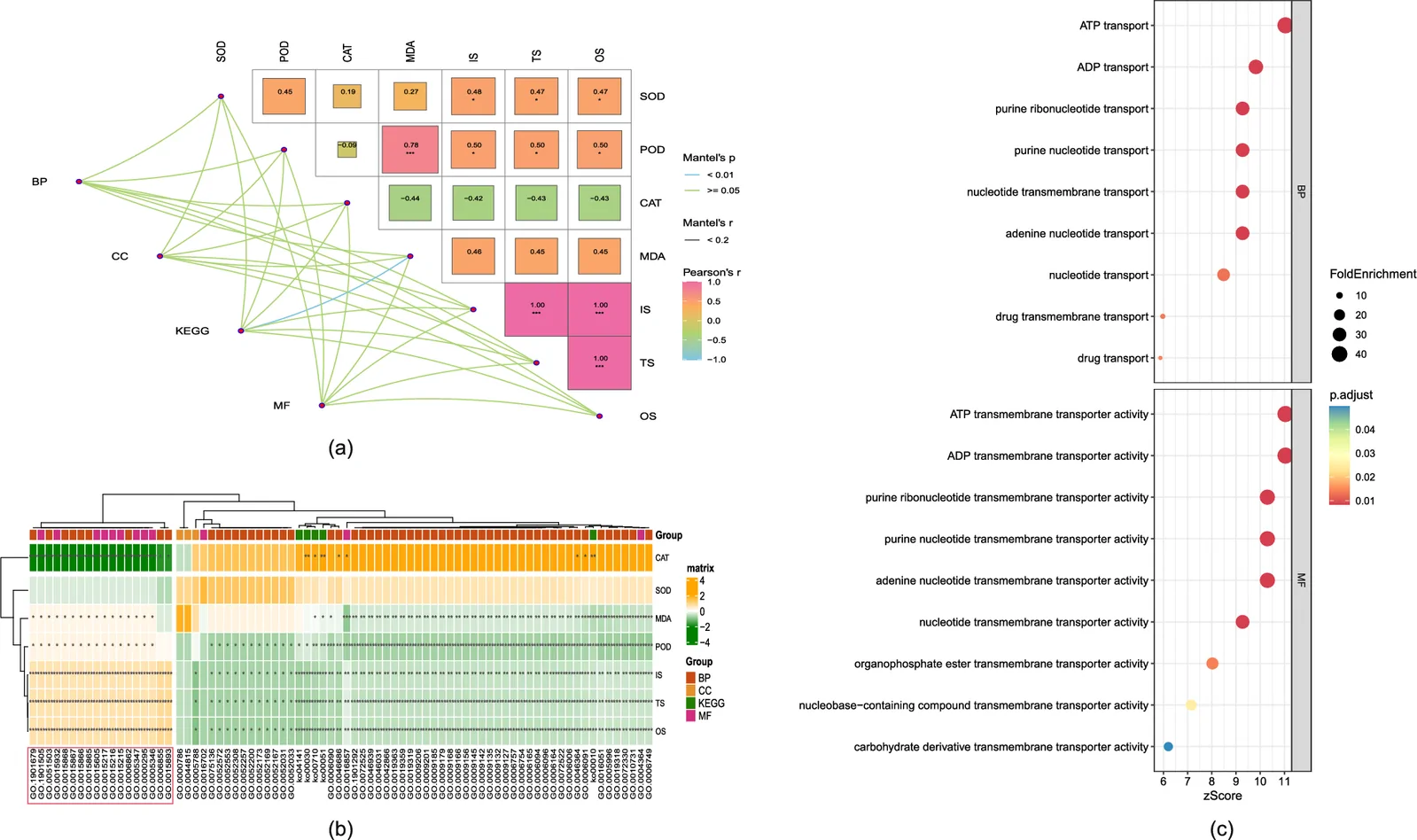
Association analysis between gene set activities and physiological variables. (**a**) Partial Mantel test results showing correlations among the four GSVA-derived gene set categories, selenium fractions, and antioxidant indices. Curve width is proportional to Mantel’s r; curve color indicates statistical significance. (**b**) Heatmap of Pearson correlations between the 18 activated GO terms and physiological variables. (**c**) Bubble chart showing the 18 terms meeting criteria of positive GSVA scores at P4/P5 and positive correlation with all three selenium fractions, with z-scores on the x-axis and bubble size proportional to gene count.

Mantel tests revealed a statistically significant overall association (Mantel’s *P* < 0.01) between all four GSVA-derived gene set activity profiles (Biological Process, Cellular Component, Molecular Function, KEGG) and the combined physiological trait matrix (antioxidant enzymes, oxidative damage markers, selenium fractions; Fig. 5a). This indicates that variation in the transcriptomic functional landscape is systematically associated with variation in measured physiological and biochemical traits, though causal directionality cannot be inferred from these data alone.

The 18 GO terms activated in the GSVA analysis were significantly and positively associated with total selenium (TS), inorganic selenium (IS), and organic selenium (OS) concentrations (Fig. 5b). Enrichment analysis of these 18 terms confirmed specific upregulation of transmembrane transport activities, particularly for nucleotides and related compounds (Fig. 5c, showing adjusted p-values and zScores). These associations suggest that nucleotide transport gene set activity tracks closely with selenium accumulation, consistent with a potential role in selenium assimilation or energy supply during this process.

At the broader level, Biological Process gene set activity was positively associated with antioxidant enzyme activities (POD, CAT, SOD), and all four gene set profiles were negatively associated with MDA content. KEGG pathway activity was associated with selenium accumulation across all three fractions (TS, IS, OS). These correlations are consistent with the possibility that selenium-induced activation of nucleotide transport pathways may support the energy requirements of antioxidant defense systems, while the concurrent reduction in primary carbon metabolism may reflect a reallocation of cellular resources toward selenium assimilation. However, these remain correlative observations; functional studies using genetic or pharmacological approaches would be required to establish causal relationships.

## Discussion

### Time-dependent selenium accumulation and physiological regulation in HK88

The progressive increase in total, inorganic, and organic selenium content in HK88 seedlings over 48 h is consistent with time-dependent accumulation patterns documented in other selenium-enriched crops^14^. The parallel rise in organic selenium fractions is nutritionally relevant, as organic selenium species, particularly selenomethionine, are generally more bioavailable to humans than inorganic selenate or selenite^27,28^. These data indicate that HK88 possesses selenium uptake and assimilation capacity under the conditions tested, which is relevant to its potential use in selenium biofortification programs^10,29^. Whether HK88 shows superior selenium accumulation relative to other soybean varieties remains to be assessed in comparative studies.

The antioxidant enzyme data revealed a staged response rather than uniform induction. At 1 h, both SOD and POD activities were lower in selenium-treated seedlings than in controls, followed by clear induction at 24 and 48 h. This biphasic pattern may indicate an initial phase of metabolic adjustment during early selenium assimilation, with enzyme-level responses following the establishment of selenium metabolism at the transcriptional level. The biphasic response of CAT was distinct: activity appeared suppressed relative to controls at 1 h, and while it recovered in both groups over time, treated plants showed lower CAT activity than controls at 48 h. This temporal separation of SOD/POD and CAT responses may reflect differential sensitivity of their respective gene promoters to selenium-induced signals^19,30–32^, or the distinct subcellular compartments in which these enzymes primarily operate. The reduction in MDA at 1 h, despite suppressed enzyme activities, suggests that the early membrane stabilization associated with selenium exposure may involve mechanisms beyond the classical enzymatic antioxidant network, a hypothesis that warrants further investigation. The convergence of MDA levels between groups by 24 and 48 h is consistent with earlier reports that selenium-mediated MDA reduction is most apparent during early to intermediate stages of treatment^33,34^.

### Transcriptional reprogramming is time-graded and converges on a stable core response

The transcriptional data showed 6,793 DEGs at 1 h, peaking at 13,196 at 24 h, then declining to 8,996 at 48 h. This pattern suggests a time-dependent response in which the most extensive reprogramming occurs at an intermediate stage, broadly consistent with transcriptional dynamics observed in other crops under selenium treatment^35,36^. It should be noted that the RNA-seq findings have not yet been independently validated by quantitative reverse transcription PCR (qRT-PCR). Future work should include qRT-PCR confirmation for at least 6–8 key DEGs, including the top up-regulated ABC transporter genes and down-regulated Calvin cycle genes, to corroborate the differential expression patterns reported here.

The 565 common DEGs maintained consistent expression differences relative to controls across all three time points, representing a stable transcriptional signature of selenium enrichment in HK88. This core set likely includes genes involved in both the initial adaptation to selenium and its longer-term metabolic integration. The enrichment of terms related to amino acid biosynthesis and plant hormone signal transduction within the common DEGs suggests that selenium may engage sulfur assimilation pathways relevant to selenoamino acid production, consistent with observations in other species^37–40^.

### Nucleotide transmembrane transport is the dominant up-regulated functional program

The most prominent functional enrichment signal among the 565 common DEGs was the coordinated up-regulation of terms related to nucleotide, purine, ATP, and ADP transmembrane transport activity. All 12 transport-related GO terms achieved significance at *p*.adjust = 8.59 × 10^−3^ or below, with zScores between 9.27 and 11.04, and were concentrated in the up-regulated gene subset. This pattern is consistent with the involvement of ABC transporters, which facilitate transmembrane transport of nucleotides, organic anions, and metabolites. ABC transporters have previously been implicated in selenium uptake and vacuolar sequestration in soybean^41,42^, and the present findings are consistent with the possibility that transcriptional up-regulation of ABC transporter-mediated nucleotide transport may contribute to the selenium accumulation phenotype observed in HK88. However, direct evidence linking these transporter genes to selenium handling in this variety would require functional validation. GSVA confirmed that nucleotide transport-related terms were selectively activated at 24 and 48 h in treated plants (mean GSVA scores of 0.663–0.828 in P4 and 0.586–0.587 in P5, versus − 0.017 to − 0.354 in Q4 and − 0.165 to − 0.303 in Q5), with the timing of activation corresponding to the period of most rapid selenium accumulation.

The enrichment of endoplasmic reticulum lumen (GO:0,005,788) as the sole significant Cellular Component term among down-regulated DEGs is also noteworthy. The ER plays roles in protein folding and in the processing of selenium-containing metabolites in plants, and the down-regulation of ER lumen-resident genes may reflect a reallocation of secretory or folding capacity in response to selenium enrichment, though this interpretation remains speculative.

### Down-regulation of primary carbon and carbohydrate metabolism under Se treatment

A consistent finding across the enrichment data was the down-regulation of genes associated with primary carbohydrate and carbon metabolism, including monosaccharide biosynthesis, gluconeogenesis, glucose and hexose metabolism, pyruvate processing, and carbohydrate biosynthesis, as well as the Calvin cycle KEGG pathway. These results suggest that selenium treatment in HK88 may suppress photosynthetic carbon assimilation and carbohydrate biosynthesis at the transcriptional level, possibly reflecting a broader reallocation of metabolic resources toward selenium uptake, organic selenium synthesis, and antioxidant capacity.

It is important to note that the selenium concentrations applied in this study yielded tissue levels of up to 0.33 mg/kg at 48 h, which appears to fall within a range that elicits stimulatory rather than phytotoxic effects, consistent with the hormesis model of selenium action whereby low-to-moderate doses may stimulate growth and antioxidant defenses, while higher doses progressively impair metabolism [17,18]. The suppression of primary carbon metabolism observed here may represent an early indicator of dose-dependent trade-offs, and whether higher selenium concentrations would further impair photosynthetic carbon fixation or compromise yield warrants investigation through dedicated dose–response studies.

The enrichment of protein glutathionylation terms also points to redox proteome remodeling under selenium influence. Glutathionylation regulates enzyme activity and protects sulfhydryl groups from irreversible oxidative modification, and its upregulation may contribute to the membrane stabilization observed at 1 h.

### Integration of transcriptomics with physiological phenotype

The Mantel test results, showing significant associations (Mantel’s *P* < 0.01) between all four GSVA-derived gene set profiles and the combined physiological phenotype, provide statistical evidence that the transcriptional changes observed are associated with, rather than independent of, selenium accumulation and antioxidant responses. The positive correlation between the 18 GSVA-activated nucleotide transport terms and all three selenium fractions (total, inorganic, organic) suggests that nucleotide transport gene set activity tracks with selenium assimilation, though whether these transport mechanisms directly facilitate selenium uptake or represent a parallel metabolic adjustment cannot be determined from correlative data alone. The association between Biological Process gene set activity and antioxidant enzyme activities (SOD, POD, CAT), together with the negative association between all gene set profiles and MDA content, offers a systems-level framework linking the transcriptional response to antioxidant physiology. These interpretations are correlative; functional studies using mutants or pathway inhibitors would be required to establish causal relationships between the identified transcriptional changes and the observed physiological outcomes.

### Contextual interpretation and comparison with previous studies

Prior investigations of selenium in soybean have focused primarily on the effects of selenium fertilization on growth parameters, selenium speciation in seeds, and isolated antioxidant responses^43,44^. The present study extends this literature in three respects. First, the time-course design combined with high-quality RNA-seq data (Q30: 97.25–98.61%; average unique mapping rate: 94.72%) captures transcriptional dynamics at 1, 24, and 48 h, revealing a hierarchical pattern of antioxidant enzyme induction that single time-point analyses would not detect. Second, the combined application of enrichment analysis, GSVA, and Mantel correlation tests provides multi-level statistical support for links between specific gene set activities and quantitative biochemical phenotypes within the same system. Third, the finding that Calvin cycle and central carbohydrate metabolism genes are down-regulated while nucleotide transmembrane transport genes are up-regulated under selenium enrichment offers a testable hypothesis: that selenium biofortification may alter the soybean transcriptome not primarily by activating stress response pathways, but by redirecting metabolic resources from primary carbon synthesis toward active transport and antioxidant defense. This hypothesis requires testing through functional genetics.

## Conclusion

This study presents a time-resolved analysis of physiological and transcriptional responses during selenium biofortification in soybean using the HK88 variety. Selenium enrichment was associated with progressive selenium accumulation (reaching 0.33 mg/kg total tissue selenium at 48 h), a staged antioxidant enzyme response, and extensive transcriptional reprogramming that peaked at 24 h with approximately 13,196 DEGs (about 18% of the annotated genome). The observed antioxidant dynamics departed from a simple stress-induction model: SOD and POD were initially lower in treated plants at 1 h and were elevated at 24–48 h, consistent with transcription-dependent activation. CAT remained comparatively suppressed throughout the treatment period, suggesting differential regulation of peroxisomal versus cytosolic antioxidant pathways. Early MDA reduction in the absence of elevated enzyme activity at 1 h is consistent with enzyme-independent membrane stabilization by selenium, though the mechanism remains to be elucidated.

Analysis of the 565 DEGs that were consistently regulated across all three time points revealed a functional polarization: up-regulated genes were associated with nucleotide and ATP/ADP transmembrane transport, while down-regulated genes were associated with primary carbon metabolism and the Calvin cycle. This pattern suggests that selenium enrichment may involve a metabolic reallocation from carbon fixation toward ATP-dependent transport and antioxidant defense, rather than a purely stress-driven response. Three ABC transporter loci showed near-perfect Pearson correlation with selenium fractions (r ≈ 1.00), identifying them as candidate genes whose roles in selenium handling warrant functional validation. The suppression of Calvin cycle genes raises the question of whether elevated selenium concentrations could impair photosynthesis, implying that optimizing selenium biofortification may require attention to the balance between selenium levels and core metabolic functions. qRT-PCR validation of key DEGs, metabolic flux analysis, and field trials are needed before these findings can be applied to soybean breeding programs.

## Supplementary Information


Supplementary Tables.
Supplementary Legends.


## Data Availability

All data generated or analyzed during this study are included in this published article. The datasets used and analyzed in the current study are available from the corresponding authors upon reasonable request. The clean reads were submitted to the sequence read archive of the National Center for Biotechnology Information (NCBI) under BioProject ID No: PRJNA1427924.
