# Supplementary material for: Selenium-induced metabolic reprogramming in soybean activates nucleotide transport pathways and suppresses primary carbon metabolism
Source: Sci Rep. 2026 Jun 16;16:27490. doi: 10.1038/s41598-026-57640-w (PMC13534699; doi:10.1038/s41598-026-57640-w)
Supplement: Supplementary file 1 [file 41598_2026_57640_MOESM2_ESM.docx]

**Supplementary Materials:** Table S1. Summary of RNA-seq raw data. Table S2. Quality control and QC analysis of RNA-seq data.
